# Microbial Diversity and Biochemical Potential Encoded by Thermal Spring Metagenomes Derived from the Kamchatka Peninsula

**DOI:** 10.1155/2013/136714

**Published:** 2013-02-27

**Authors:** Bernd Wemheuer, Robert Taube, Pinar Akyol, Franziska Wemheuer, Rolf Daniel

**Affiliations:** ^1^Department of Genomic and Applied Microbiology and Goettingen Genomics Laboratory, Institute of Microbiology and Genetics, Georg-August-University Goettingen, Grisebachstraße 8, 37077 Goettingen, Germany; ^2^Section of Agricultural Entomology, Department for Crop Sciences, Georg-August-University Goettingen, Grisebachstraße 6, 37077 Goettingen, Germany

## Abstract

Volcanic regions contain a variety of environments suitable for extremophiles. This study was focused on assessing and exploiting the prokaryotic diversity of two microbial communities derived from different Kamchatkian thermal springs by metagenomic approaches. Samples were taken from a thermoacidophilic spring near the Mutnovsky Volcano and from a thermophilic spring in the Uzon Caldera. Environmental DNA for metagenomic analysis was isolated from collected sediment samples by direct cell lysis. The prokaryotic community composition was examined by analysis of archaeal and bacterial 16S rRNA genes. A total number of 1235 16S rRNA gene sequences were obtained and used for taxonomic classification. Most abundant in the samples were members of *Thaumarchaeota*, *Thermotogae*, and *Proteobacteria*. The Mutnovsky hot spring was dominated by the Terrestrial Hot Spring Group, *Kosmotoga*, and *Acidithiobacillus*. The Uzon Caldera was dominated by uncultured members of the Miscellaneous Crenarchaeotic Group and *Enterobacteriaceae*. The remaining 16S rRNA gene sequences belonged to the *Aquificae*, *Dictyoglomi*, *Euryarchaeota*, *Korarchaeota*, *Thermodesulfobacteria*, *Firmicutes*, and some potential new phyla. In addition, the recovered DNA was used for generation of metagenomic libraries, which were subsequently mined for genes encoding lipolytic and proteolytic enzymes. Three novel genes conferring lipolytic and one gene conferring proteolytic activity were identified.

## 1. Introduction

Sites of volcanic activity can be found all over the world and even under the sea. Volcanic regions provide a variety of different environments for extremophilic archaeal and bacterial microorganisms. Well-known examples of such extreme environments are terrestrial surface hot springs. With respect to geographical, physical, environmental, and chemical characteristics, hot springs are unique sites for extremophilic microorganisms [1–3]. Extremophiles inhabiting hot springs are considered to be the closest living descendants of the earliest life forms on Earth [4, 5]. Therefore, these springs provide insights into the origin and evolution of life. In addition, thermophiles and hyperthermophiles produce a variety of hydrolytic enzymes such as lipases, glycosidases, peptidases and other biomolecules, which are of industrial interest [6–8]. For example, Hotta et al. [9] found an extremely stable carboxylesterase in the hyperthermophilic archaeon *Pyrobaculum calidifontis* VA1, and Arpigny et al. [10] identified a novel heat-stable lipolytic enzyme in *Sulfolobus acidocaldarius* DSM 639.

Especially in extreme environments, most microorganisms are reluctant to cultivation-based approaches [11, 12]. Therefore, culture-independent metagenomic strategies are promising approaches to assess the phylogenetic composition and functional potential of microbial communities living in extreme environments [7, 13, 14]. For example, Simon et al. studied the prokaryotic community in glacier ice and found a highly diverse bacterial community [15]. In 1998, Hugenholtz et al. [1] investigated the bacterial diversity in the Obsidian Pool in Yellowstone National Park and identified several new bacterial candidate divisions. The same pool and two others were studied later by Meyer-Dombard et al. [16]. They encountered diverse bacterial and archaeal communities in all three hot springs.

In the present study, we investigated the phylogenetic composition and metabolic potential of two microbial communities derived from two extreme sites of the Kamchatka peninsula, which is located in the Far East of Russia. The Kamchatka peninsula comprises an area of approximately 472,300 km^2^ and is described as the *land of fire *by its first explorers due to the high density of volcanoes and associated volcanic phenomena. For example, the largest active volcano of the northern hemisphere, the Klyuchevskaya Sopka, is located on the Kamchatka Peninsula. Sediment samples analyzed in this study were taken from two hot springs providing a thermoacidophilic (70°C, pH3.5–4) or a thermophilic (81°C, pH7.2–7.4) environment. The composition of the prokaryotic communities of the two Kamchatkian hot springs was assessed by 16S rRNA gene analysis. In addition, metagenomic libraries were generated and screened for novel biocatalysts. 

## 2. Materials and Methods

### 2.1. Sampling and DNA Extraction

Two sediment samples were taken from the hot springs located on the Kamchatka peninsula in summer 2001. The first sample was collected from a thermoacidophilic spring (70°C, pH3.5–4) at the Mutnovsky volcano (52.453 N, 158.195 E). The second sample was taken from a thermophilic spring (81°C, pH 7.2–7.4) in the Uzon Caldera (54.5 N, 159.967 E). The chemical analysis of both sediment samples is shown in Table 1.

DNA was extracted as described by Zhou et al., 1996 [17]. The concentration of the recovered DNA was quantified using a NanoDrop ND-1000 spectrophotometer (PEQLAB, Erlangen, Germany).

### 2.2. Amplification of 16S rRNA Genes and Generation Clone Libraries

To assess the prokaryotic community structure, archaeal and bacterial 16S rRNA genes were amplified by PCR and analyzed. The PCR reaction mixture (50 *μ*L) contained 2.5 *μ*L of 10-fold Mg-free *Taq* polymerase buffer, 200 *μ*M of each of the four deoxynucleoside triphosphates, 1.75 mM MgCl_2_, 0.4 *μ*M of each primer, 1 U of *Taq* DNA polymerase (Fermentas, St. Leon-Rot, Germany), and approximately 25 ng of recovered DNA as a template. Prokaryotic 16S rRNA genes were amplified with the following set of primers: 8F 5′-AGAGTTTGATCMTGGC-3′ [18] and 1114R 5′-GGGTTGCGCTCGTTRC-3′ [19], A800F 5′-GTAGTCCYGGCYGTAAAC-3′ [20] and A1530R 5′-GGAGGTGATCCAGCCG-3′ [21], and Arch8F 5′-TCCGGTTGATCCTGCCGG-3′ [15] and Arch958R 5′-YCCGGCGTTGAMTCCAATT-3′ [22]. The following thermal cycling scheme was used: initial denaturation at 94°C for 2 min, 25 cycles of denaturation at 94°C for 1.5 min, annealing at 56°C (8F and 1114R), 51°C (A800F and A1530R), or 55°C (Arch8F and 958R), followed by extension at 72°C (1 min for 1 kb). The final extension was carried out at 72°C for 10 min. Negative controls were performed by using the reaction mixture without template. The obtained PCR products were purified using the Wizard SV Gel and PCR clean up system (Promega, Madison, USA) and subsequently cloned into pCR2.1-TOPO as recommended by the manufacturer (Invitrogen, Carlsbad, USA). The resulting recombinant plasmids were used to transform *Escherichia coli* TOP 10 cells. A total of 1271 insert-carrying plasmids were isolated from randomly selected *E. coli* clones. The insert sequences were determined by the Göttingen Genomics Laboratory (Göttingen, Germany).

### 2.3. Analysis of 16S rRNA Genes

To assess the prokaryotic community structure, the retrieved 16S rRNA gene sequences were analyzed using QIIME [23]. The obtained 16S gene sequences were edited using gap4 [24] and initially checked for the presence of chimeric sequences using Mallard [25], Bellerophon [26], and Chimera Check [27]. Remaining sequences were clustered employing the UCLUST algorithm [28] and the following QIIME scripts: pick_otus.py and pick_rep_set.py. The sequences were clustered in operational taxonomic units (OTUs) at 1, 3, and 20% genetic dissimilarity.

The phylogenetic composition of the prokaryotic communities in both samples was determined using the QIIME assign_taxonomy.py script. A BLAST alignment [29] against the most recent SILVA ARB database [30] was performed. Sequences were classified with respect to the taxonomy of their best hit in the ARB database. Finally, OTU tables were generated. Rarefaction curves, Shannon indices [31], and Chao1 indices [32] were calculated employing QIIME. In addition, the maximal number of OTUs (*n*_max⁡_) was estimated for each sample using the Michaelis-Menten-fit alpha diversity metrics included in the QIIME software package.

One sequence per OTU (1% genetic distance) was further used for the construction of phylogenetic trees. Sequences were imported into the most recent SSU Ref SILVA database of the ARB program package [33]. Multiple sequence alignments were checked manually and improved by employing the ARB editor tool. Phylogenetic trees were created by employing the maximum parsimony algorithm implemented in ARB. The robustness of obtained tree topologies was evaluated by bootstrap analysis with 100 resemblings.

### 2.4. Construction of Small-Insert Metagenomic Libraries

To exploit the biochemical potential, metagenomic small-insert libraries were generated. Due to the low DNA recovery, starting material for the generation of these libraries was obtained by multiple displacement amplification employing the GenomiPhi V2 DNA Amplification Kit (GE Healthcare, Munich, Germany). To improve cloning efficiency, hyperbranched structures were resolved and the DNA was inserted into pCR-XL-TOPO (Invitrogen) as described by Simon et al. [34]. In this way, two metagenomic libraries were generated.

### 2.5. Screening for Hydrolytic Activity and Identification of Corresponding Genes

The constructed metagenomic libraries were screened for genes conferring lipolytic or proteolytic activity using a function-driven approach. The constructed libraries were used to transform *E. coli* DH5*α* cells. Recombinant cells were plated on LB agar plates containing either 1% tributyrin (lipolytic activity) [35] or 2% skim milk (proteolytic activity) [36]. Plates were incubated at 37°C for up to two weeks. Hydrolytic activity is indicated by halo formation. To determine the substrate specificity of the protease-producing clones, skim milk was replaced by 0.3% (w/v) azocasein, azoalbumin, or elastin-Congo red. Insert sequences of recombinant plasmids derived from positive clones were determined by the Göttingen Genomics Laboratory. The retrieved insert sequences were edited employing gap4 [24], and putative ORFs were annotated using Artemis (version 11.0) [37].

### 2.6. Cloning of Genes Conferring Lipolytic or Proteolytic Activity into Expression Vectors and Purification of the Corresponding Gene Products

For enzyme production of lipolytic proteins, identified genes were cloned into pET101-TOPO according to the Champion pET101 directional TOPO Expression Kit (Invitrogen). In this way, sequences encoding a His_6_ tag and a V5 epitope provided by the vector were added to the 3′ end of the coding regions. Alternative start codons were replaced by ATG. *Escherichia coli* BL21 Star (DE3) (Invitrogen) was used as a host for enzyme production. The production was performed as recommended by the manufacturer. Subsequently, recombinant cells were harvested by centrifugation, washed with Tris buffer (30 mM, pH 8.0), and resuspended in 50 mmol L^−1^ of sodium phosphate buffer containing 0,3 mol L^−1^ NaCl (pH 8.0). The cells were disrupted employing a French Press (1.38 × 10^8^ Pa). Subsequently, the extract was cleared by centrifugation at 14,000 g and 4°C for 45 min. The supernatant was used as a source for soluble proteins. Recombinant proteins were purified using the Protino Ni-TED 2000 packed columns (Macherey-Nagel, Düren, Germany) as recommended by the manufacturer. Protein preparations were dialyzed using sodium phosphate buffer (50 mM, pH 7.5) to remove residual imidazole. Protein concentration in the purified sample was determined with Roti-Quant (Carl Roth, Karlsruhe, Germany) as suggested by the manufacturer. The purity of the protein preparations was analyzed by SDS-polyacrylamide gel electrophoresis according to Laemmli [38].

 For protein production of the putative protease, the corresponding gene (*pepBW1*) was cloned into pBAD/Myc-His (Invitrogen) using a modified fusion method [39]. The gene was amplified in two PCR reactions with the following two sets of primer pairs containing synthetic sites (underlined) for cloning into the vector: pair 1, 5′-CATGGTGTTCAATAAATATGTCTT-3′ and 5′-CTAGGGTAGACTTAACGC-3′ and pair 2, 5′-GTGTTCAATAAATATGTCTTATT-3′ and 5′-CGCTAGGGTAGACTTAACGC-3′. After mixing, denaturation, and hybridization, four different hybridization products are formed of which one contained overhangs complementary to vector digested with *Nco*I (Fermentas) and *Bsp*119I (Fermentas). The preligation mixture (10 *μ*L) contained 1 *μ*L O-buffer (Fermentas) and approximately 50 ng of each PCR product. To create the appropriate overhangs for cloning, the following thermal cycling scheme was used: denaturation at 95°C for 3 min, 4 cycles of reannealing at 65°C for 2 min, and reannealing at 25°C for 15 min. The ligation reaction mixture (20 *μ*L) contained the preligation mixture, 1 *μ*L of O-Buffer (Fermentas), 1 *μ*L ATP (10 mM), 1 U of T4 DNA ligase, and 10 ng of pBAD/Myc-His digested with *Nco*I and *Bsp*119I. The reaction was incubated at 16°C overnight and inactivated by heating at 65°C for 10 min. The resulting recombinant plasmids were then used to transform *E. coli* top 10 cells.

### 2.7. Characterization of Lipolytic Activity

Determination of enzyme specificity against different triacylglycerides was determined by growing *E. coli* BL21 (DE3) harboring the recombinant plasmids on LB agar plates containing triacylglycerides with different chain length (C4 to C18). Plates were supplemented with IPTG to a final concentration of 0.1 mM to induce gene expression.

For quantitative analysis, *p*-nitrophenyl esters with various chain lengths were used as descripted by Rashamuse et al. [40]. Routine esterase activity assays were performed by measuring the release of *p*-nitrophenol from a *p*-nitrophenyl (*p*-NP) ester at 410 nm using a Cary 100 UV-Vis spectrophotometer (Varian, Palo Alto, USA) with a Peltier temperature controller. Unless otherwise described, enzyme activity was measured at 50°C in Tris-HCl (50 mmol L^−1^; pH 7.5) with *p*-NP caprylate (1 mmol L^−1^; dissolved in 2-propanol) as a substrate. *p*-NP caprylate was used as a substrate in the standard assay because of its stability at high temperatures and alkaline pH values. Enzyme activity of EstBW2 was determined with *p*-NP butyrate as the enzyme showed no activity with the other substrates tested. Therefore, temperature and pH dependence of EstBW2 were not measured above 75°C and pH 8. All measurements were performed in triplicate.

To determine substrate specificity, enzyme activity was measured at standard assay conditions employing the following *p*-NP esters of various chain lengths: *p*-NP acetate (C2), *p*-NP butyrate (C4), *p*-NP caprylate (C8), *p*-NP caprate (C10), *p*-NP laurate (C12), and *p*-NP palmitate (C16). 

The temperature dependence of enzyme activity was determined between 20 and 95°C under standard assay conditions. To compensate temperature effects on pH values, buffers were preheated to set-point temperature and adjusted using Tris buffer (50 mmol L^−1^). Thermostability was measured by incubating the enzyme at different temperatures over various time periods. Enzyme activity was subsequently measured under standard assay conditions. Optimal pH values for enzyme activity were measured under standard assay conditions employing different overlapping buffer solutions (50 mM): sodium acetate buffer (pH 4 and 5), sodium phosphate buffer (pH 5, 6, and 7), Tris-HCl (pH 7, 8, and 9), CHES (pH 9 and 10), and CAPS (pH 10 and 11).

The effect of different detergents on enzyme activity was determined by under standard assay conditions in the presence of 1 mM AgNO_3_, 1 mM CaCl_2_, 1 mM CoCl_2_, 1 mM cetyltrimethylammonium bromide (CTAB), 1 mM CuCl_2_, 1 mM ethylenediaminetetraacetic acid (EDTA), 1 mM FeCl_3_, 1 mM KCl, 1 mM MgCl_2_, 1 mM MnCl_2_, 1 mM NaCl, 1 mM NiSO_4_, 1 mM Sodium Dodecyl Sulfate (SDS), 1 mM ZnCl_2_, 0.01% (v/v) Tween 80, or 0.01% (v/v) 2-Mercaptoethanol. The serine dependence of the recovered lipolytic enzymes was validated under standard assay conditions by incubation in the presence of 1 mM phenylmethylsulfonyl fluoride (PMSF). In addition, we analyzed the significance of the determined effects on enzyme activity. As the enzyme tests were performed in triplicate, we assumed that all measured enzyme activities were normally distributed. The variance homogeneity was tested employing the *F*-test, and the significance of the detergent effects was subsequently tested either with the Student's *t*-test (homogenous variances) or the Welch's *t* test (heterogeneous variances). All statistical analyses were performed in *R* [41].

### 2.8. Nucleotide Sequence Accession Numbers

The 16S rRNA gene sequences have been deposited in GenBank under accession numbers HM149792–HM150618. Nucleotide sequences of the four identified genes have been deposited in GenBank under the accession numbers HM063743 (plpBW1), HM063744 (estBW1), HM063745 (estBW2), and HM063746 (pepBW1).

## 3. Results

### 3.1. Sampling and Chemical Properties of the Investigated Sediments

Sediment samples were collected from two Kamchatkian hot springs. The springs were located near the Mutnovsky volcano (Mutnovsky sample) and in the Uzon Caldera (Uzon sample), which represent a thermoacidophilic (70°C, pH 3.5–4) and a thermophilic (81°C, pH 7.2–7.4) environment, respectively. Both investigated sediments were chemically distinct from each other (Table 1). The Mutnovsky sample contained higher Al, Ca, Co, Cu, Fe, Pb, and Zn concentrations than the Uzon sample. For As, B, Ba, K, Mn, and Na concentrations, the opposite was recorded. The concentrations of Cr, Mg, Ni, Sr, P, Ti, and V and the total organic carbon contents were almost identical in both samples.

### 3.2. Isolation of Metagenomic DNA and Construction of Metagenomic Libraries

To assess the prokaryotic diversity and metabolic potential by metagenomic approaches, environmental DNA was extracted from both samples. Approximately, 2.7 *μ*g DNA per 10 g sediment was recovered from both samples. After removal of remaining salts, archaeal and bacterial 16S rRNA genes were amplified from the purified DNA. The resulting PCR products were used for the generation of 16S rRNA gene libraries. A total of 1271 clones were sequenced from these libraries. After quality filtering and removal of potential chimeric sequences, 1235 high-quality 16S rRNA gene sequences were obtained (536 for the Mutnovsky sample, 699 for the Uzon sample). The DNA from both samples was also used to construct metagenomic libraries. The Mutnovsky library comprised approximately 479,000 plasmids with an average insert size of 5.3 kb. The percentage of insert-carrying plasmids was 74%. The Uzon library consisted of approximately 117,000 plasmids with an average insert size of 4 kb. The percentage of insert-carrying plasmids was 85%. In summary, the generated small-insert metagenomic libraries harbored approximately 2.27 Gbp of cloned environmental DNA.

### 3.3. Archaeal Community Structures

**Figure 1:**
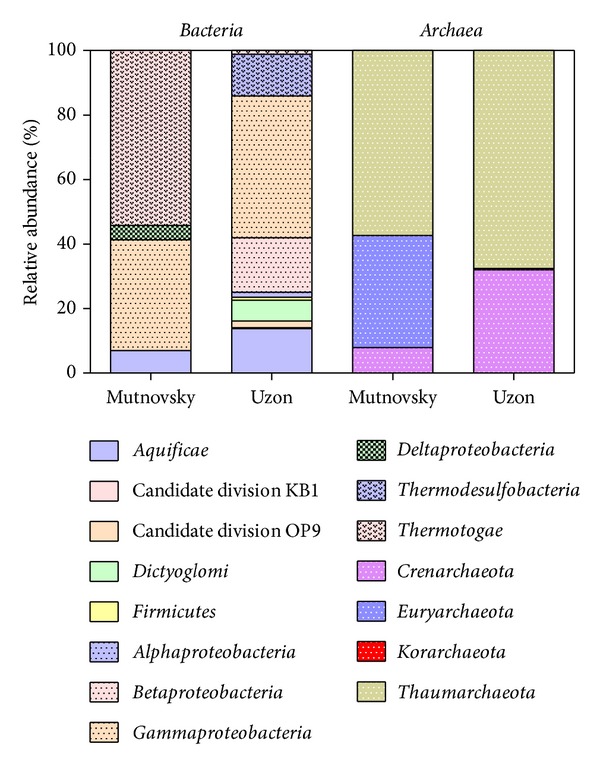
Relative sequence abundances of different archaeal and bacterial phyla and proteobacterial classes.

We were able to assign 265 16S rRNA gene sequences of both samples to the domain *Archaea*. The classified sequences were affiliated to four different archaeal phyla (Figure 1). The *Thaumarchaeota* was the most abundant archaeal phylum in both samples (57% and 68% of all sequences, resp.). Most of the sequences were affiliated to Miscellaneous Crenarchaeotic Group nowadays belonging to the recently proposed *Thaumarchaeota* (37%). Another abundant thaumarchaeotic group was the Terrestrial Hot Spring Group (24.7%). The majority of the remaining sequences of the Mutnovsky sample were affiliated to uncultured members of the *Euryarchaeota* (34.7%), which were only detected in this sample. The majority of the remaining Uzon sequences belonged to the *Crenarchaeota* (32.1%). Sequences were affiliated to known genera such as *Sulfophobococcus* (12.1%), *Thermofilum* (6.8%), *Ignisphaera* (5.3%), and to *Desulfurococcus kamchatkensis* (4.2%). The archaeal phylum *Korarchaeota* was only identified in the Uzon sample (1 sequence).

### 3.4. Diversity and Species Richness of Archaeal Communities

To determine the archaeal diversity and richness, rarefaction analyses were performed with QIIME. The observed OTU numbers in the Mutnovsky sample and the Uzon sample were 33 and 13 (1% genetic distance), 25 and 11 (3% genetic distance), and 7 and 5 (20% genetic distance), respectively (Table 2). The maximal expectable number of clusters for both samples was determined based on the Michaelis-Menten_fit metrics. On average, more than 90% of the entire archaeal community was covered by the surveying effort. Shannon indices of the Mutnovsky and Uzon sample were 1.83 and 2.96 (1% genetic distance), 2.70 and 1.83 (3% genetic distance), and 0.86 and 1.87 (20% genetic distance), respectively. These indicated low archaeal diversity in the investigated samples. Comparison of the rarefaction analyses with the number of OTUs determined by Chao1 richness estimator revealed that, at 1 and 3% genetic distance, the rarefaction curves were almost saturated (Figure 4). Thus, the majority of the estimated richness was recovered by the surveying effort (Table 2).

### 3.5. Bacterial Community Structures

We were able to assign 271 sequences for Mutnovsky and 434 sequences for Uzon sample to the domain *Bacteria*. The classified sequences were affiliated to three and eight different bacterial phyla and candidate divisions in the Mutnovsky sample and the Uzon sample, respectively (Figure 1). The *Thermotogae* was the most abundant bacterial phylum in the Mutnovsky sample (54%). This phylum was almost absent in the Uzon sample (1%). Interestingly, all sequences in the Mutnovsky sample were further affiliated to uncultured members of the genus *Kosmotoga*. This genus was completely absent in the Uzon Caldera. The *Proteobacteria* were the second most abundant phylum in the Mutnovsky sample (39%) and the most abundant one in the Uzon Caldera sample (62%). Most of these sequences were further assigned to *Acidithiobacillus caldus *ATCC 51756 (28%) in the Mutnovsky sample and different genera within the *Enterobacteriacaeae* (41%) in the Uzon sample. The *Aquificae* were the third most abundant phylum in the Uzon sample (13.4%). The corresponding sequences were assigned to *Sulfurihydrogenibium rodmanii* (9.7%) and *Thermosulfidibacter takaii *(2.8%) and uncultured members of the *Aquificae*. Another abundant bacterial phylum was  *Thermodesulfobacteria* (12.9%). All the sequences belonged to the genus *Caldimicrobium*. The remaining sequences were affiliated to *Dictyoglomus thermophilum* and *Dictyoglomus turgidum* of the *Dictyoglomi* (6.5%), the Candiate division OP9 (2.1%), the *Firmicutes* (0.9%), and the Candidate division KB1 (0.2%).

### 3.6. Diversity and Species Richness of Bacterial Communities

The observed OTU numbers in both hot springs were 17 and 50 (1% genetic distance), 12 and 42 (3% genetic distance), and 10 and 11 (20% genetic distance) in the Mutnovsky sample and the Uzon sample, respectively (Table 2). Analysis of the maximal expectable number of clusters indicated that more than 94% of the entire bacterial community was recovered by the surveying effort. Correspondingly, comparison of the rarefaction analyses with the number of OTUs determined by Chao1 richness estimator revealed that at 1%, 3%, and 20% genetic distance, the rarefaction curves were almost saturated (Table 2, Figure 4).

### 3.7. Screening of Metagenomic Libraries

The two generated metagenomic libraries were employed in a function-based screening to identify novel lipolytic and proteolytic enzymes. Three novel genes encoding lipolytic enzymes (*plpBW1*, *estBW1*, and *estBW2*) and one gene encoding a proteolytic enzyme (*pepBW1*) were identified during the screening of the metagenomic library derived from the Uzon sample. No hydrolytic enzymes were identified within the metagenomic library derived from the Mutnovsky sample. 

The closest relatives of all identified protein sequences originated from known thermophiles. They were similar to uncharacterized putative gene products derived from *Desulfurococcus kamchatkensis* (PepBW1), *Sulfurihydrogenibium azorense *(PlpBW1 and EstBW2), and *Thermobaculum terrenum *(EstBW1) (Table 3).

PlpBW1 was affiliated to the patatin-like proteins (PLPs). Four conserved domains are described for this enzyme type [42], which could all be identified within the amino acid sequence (data not shown). Interestingly, PLPs do not possess a catalytic triad. The lipolytic activity is conferred by a catalytic dyad formed by a serine residue and an aspartate residue [42]. EstBW1 and EstBW2 were affiliated to family V of lipolytic enzymes according to the classification system of Arpigny and Jaeger [43]. PepBW1 was classified employing the MEROPS database [44]. It was affiliated to the subtilisin family (family S8). Interestingly, the *pepBW1* gene sequence was almost identical to that of a putative gene encoding a serine peptidase of *Desulfurococcus kamchatkiensis* (Table 3);* Desulfurococcus kamchatkiensis *belongs to the *Crenarchaeota *and was also isolated from a thermal spring within the Uzon Caldera [45]. In addition, the 16S rRNA gene sequence of this species was found in our 16S analysis of the Uzon sample.

### 3.8. Characterization of Recombinant Enzymes

To characterize all recombinant proteins, the genes conferring lipolytic and proteolytic activity were cloned into expression vectors. The recombinant *E. coli* strain containing PepBW1 was tested towards different proteins and showed proteolytic activity with skim milk and elastin-Congo red but not with azoalbumin or azocasein.

The activities of the recombinant lipolytic proteins were tested towards different triacylglycerides. All proteins showed activity with tributyrin as substrate. In addition, PlpBW1 showed activity with long-chain triacylglycerides, up to trimyristin (C14). Hydrolysis of different *p*-nitrophenyl esters was used to further analyze the substrate specificity (Figure 5(a)). PlpBW1 and EstBW1 showed highest activity with *p*-NP acetate and *p*-NP butyrate, respectively. Both enzymes exhibit activity towards all tested *p*-NP esters, except *p*-NP palmitate. The activity decreased with increasing chain length. In contrast, EstBW2 showed only activity towards *p*-NP butyrate. Specific activities under standard assay conditions using the optimal substrate were 2.6 ± 0.3 U/mg (PlpBW1), 2.33 ± 0.32 U/mg (EstBW1), and 1.89 ± 0.21 U/mg (EstBW2). Based on the results, all three lipolytic enzymes are most likely carboxylesterases and not lipases.

All lipolytic enzymes were active over a wide temperature range. PlpBW1, EstBW1, and EstBW2 retained a minimum of 50% activity from 60 to 90°C, 65 to 95°C, and 40 to 75°C, respectively (Figure 5(b)). Maximal activities were recorded for PlpBW1 at 85°C, for EstBW1 at 90°C, and for EstBW2 at 65°C. We further determined the stability of the three lipolytic enzymes with respect to different temperatures. The half-lives of PlpBW1 were 45 min at 70°C, 15 min at 80°C, and 5 min at 90°C. EstBW1 exhibited half-lives of 5 h at 70°C, 2.5 h at 80°C, and a remarkable half-life of 15 min at 90°C. EstBW2 was less stable at 90°C (7 min half-live), but the activity was almost unaffected by 5 h of incubation at 70°C and 80°C (data not shown).

The pH effect on enzyme activity was measured at pH values ranging from 4 to 11 (Figure 5(c)). All enzymes exhibited high activity at neutral or alkaline pH values. Maximal activities were determined at pH 10 (PlpBW1) and pH 7 (EstBW1 and EstBW2). 

Addition of EDTA, KCl, or NaCl to the reaction mixture had no significant effect on enzyme activity (*P* > 0.05), whereas all other tested detergents exhibited an effect on the activity of at least one of the recovered enzymes (Table 4). CTAB, Tween 80, and ZnCl_2_ impacted the activities of all three enzymes significantly (*P* < 0.05). PlpBW1 showed a more than 2.5-fold higher activity in presence of CTAB, whereas EstBW1 and EstBW2 displayed a loss in activity. Tween 80 increased enzyme activity of PlpBW1 and EstBW2 but not that of EstBW1. The addition of ZnCl_2_ decreased the activity of all three recombinant enzymes. Lipolytic activity of EstBW1 and EstBW2 was completely inhibited by the phenylmethylsulfonyl fluoride, indicating the presence of a serine residue at the active site of both enzymes. Interestingly, the activity of PlpBW1 was not affected by PMSF.

## 4. Discussion

### 4.1. Prokaryotic Community Composition in the Kamchatkian Springs

The number of metagenomic studies has been rapidly increased over the past years. Metagenomics has been employed to assess and exploit the biodiversity of many habitats including environments of extremophiles [1, 15, 16, 46, 47]. In this study, we investigated the prokaryotic diversity of two hot springs located on the Kamchatka peninsula. We found different bacterial and archaeal communities at both sites, which were dominated by *Proteobacteria*, *Thermotogae*, and *Thaumarchaeota*. 

Jackson et al. (2001) studied a mat derived from the Norris Geyser Basin, an acidic thermal spring in the Yellowstone National Park [46]. They found community pattern comparable to that in the Mutnovsky sample with one difference. They were not able to identify *Thaumarchaeota*, which is not surprising as this phylum was first proposed in 2008 [48]. Members of this phylum are not restricted to thermophilic habitats as they were originally described as mesophilic *Crenarchaeota* [48–50]. A study by Meyer-Dombard et al. (2005) investigated the prokaryotic community in three thermal springs in the Yellowstone National Park (the Silvan Spring, the Bison Pool, and the Obsidian Pool) [16]. Whereas the other pools have a rather neutral milieu, the Silvan Spring has a low pH of 5. However, the prokaryotic community structure of this acidic spring was different to that found in the acidic Mutnovsky spring sample. Meyer-Dombard et al. identified the *Crenarchaeota* as the most abundant archaeal group, whereas *Thaumarchaeota* were the most abundant group in our study. A more recent study on prokaryotic community composition of hot springs on the Tibetan Plateau also found *Thaumarchaeota* as the dominant archaeal group [48].

**Figure 2:**
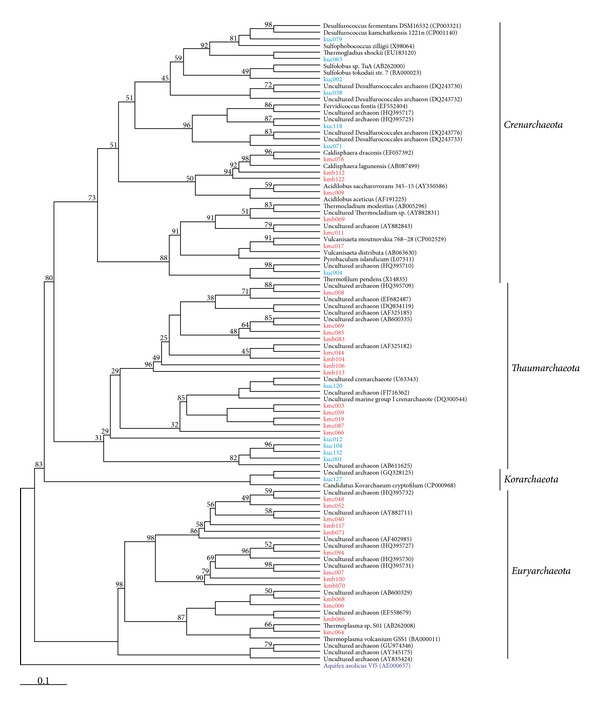
Maximum parsimony phylogenetic tree based on all archaeal 16S rRNA gene sequences. The tree was calculated using the ARB software package [33]. Sequences are characterized by sample designation (km, Kamchatka Mutnovsky; ku, Kamchatka Uzon Caldera), length of amplicon ((b), 730 bp; (c), 950 bp), number of sequence, and accession number. Sequences derived from the Mutnovsky sample are shown in red and those from the Uzon sample in blue. Numbers at branch nodes are bootstrap values (only values ≥25 are shown). The tree is rooted with the 16S rRNA gene sequence of *Aquifex aeolicus* Vf5 as an outgroup.

**Figure 3:**
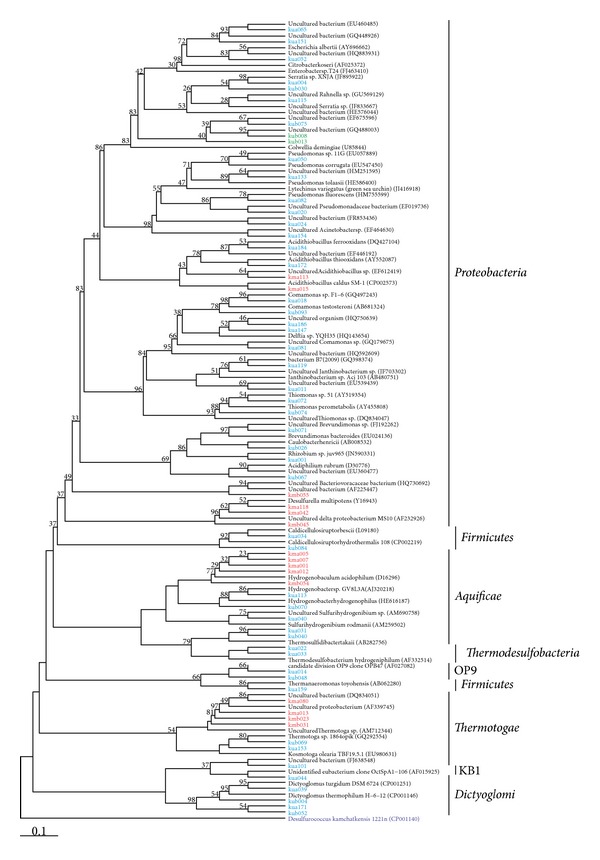
Maximum parsimony phylogenetic tree based on all bacterial 16S rRNA gene sequences. The tree was calculated using the ARB software package [33]. Sequences are characterized by sample designation (km, Kamchatka Mutnovsky; ku, Kamchatka Uzon Caldera), length of amplicon ((a), 1100bp, (b), 730bp), number of sequence, and accession number. Sequences derived from the Mutnovsky are shown in red and those from the Uzon Caldera in blue. OTUs shared by both sites are depicted in green. Numbers at branch nodes are bootstrap values (only values ≥25 are shown). The tree is rooted with the 16S rRNA gene sequence of *Desulfurococcus kamchatkensis *1221n as an outgroup.

**Figure 4:**
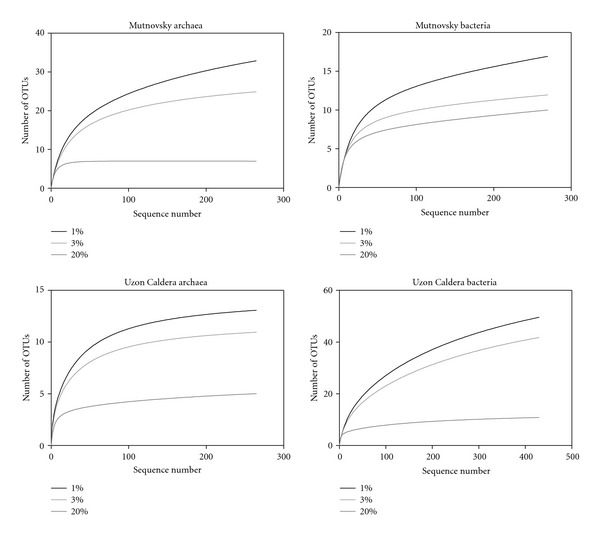
Rarefaction curves for both sampling sites. Curves were calculated at 1%, 3%, and 20% genetic distance level employing QIIME [23].

**Figure 5:**
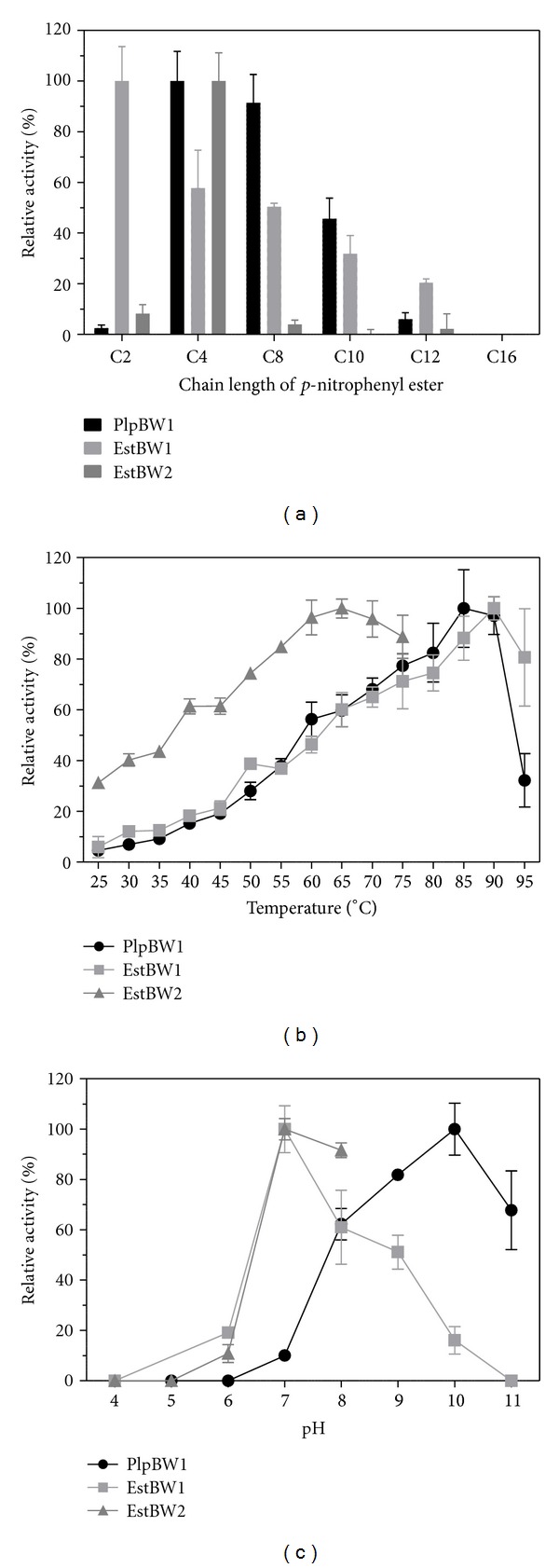
Relative activities of the three lipolytic enzymes towards different *p*-nitrophenyl esters with various chain lengths (a), at different temperatures (b), and pH values (c). The activity of estBW2 could not be measured above 75°C and pH 8 due to the instability of the *p*-NP butyrate under these conditions.

 Analysis of the Uzon sample revealed a more diverse prokaryotic community than in the Mutnovsky sample. Only two OTUs at 1% genetic distance were shared, whereas all the other OTUs were unique for each sample (Figures 2 and 3). The observed differences in community composition between the two sampling sites might be due to the different temperatures and pH values at the sites. However, Huang et al. (2011) found no statistical correlation between temperature and diversity [48]. 

Despite the geographical separation, the Obsidian Pool and the Uzon Caldera hot spring share a very similar community structure, as almost the same dominant archaeal and bacterial groups were identified [16]. In addition, some rare phyla were present in both samples, that is, the *Korarchaeota*. This phylum is a relatively new phylum first described by Barns et al. in 1996 [51]. Another rare bacterial group found in both samples was the Candidate division OP9. These results confirm also the presumption proposed for other extreme environments that similar environmental conditions result in similar microbial communities [15]. 

### 4.2. Hydrolytic Enzymes

In the present study, we were able to identify three novel lipolytic enzymes and one proteolytic enzyme. The determined optimal temperatures and pH values reflect the environmental conditions of the samples used for DNA isolation, indicating that the environment shapes the characteristics of the enzymes. Correspondingly, the characterized lipolytic enzymes (PlpBW1, EstBW1, and EstBW2) showed features similar to those of other metagenome-derived esterases, which were identified in thermophilic sites. Rhee et al. (2005) identified a thermophilic esterase in metagenomic libraries generated from hot spring and mud hole DNA [52]. The enzyme was active from 30 to 95°C and exhibited an optimal pH value of approximately 6.0. Tirawongsaroj et al. (2008) screened metagenomic libraries derived from a Thailand hot spring and identified two novel lipolytic enzymes, of which one was also characterized as a patatin-like protein [53]. To our knowledge, PlpBW1 is the second reported patatin-like protein derived from a hot spring metagenomic library up to now [53]. In contrast to most other lipolytic enzymes containing a serine residue in the active site, PlpBW1 is not inactivated by the inhibitor PMSF [51, 54]. The effect of Zn^2+^ ions recorded for all recombinant enzymes investigated in this study was also mentioned for esterases studied by Chu et al. [54]. They also recorded a decrease of activity in presence of Zn^2+^ ions. The activity of the recovered lipolytic enzymes was positively or negatively influenced by addition of CTAB or Tween 80 (Table 4). It has been shown that these detergents can either promote or decrease activity of lipolytic enzymes by formation of micellar aggregates and monomers which then interact with hydrophobic parts of the enzymes [55].

In addition to the esterases, metagenomic libraries were mined for proteolytic activity. The identified serine peptidase, PepBW1, is the first metagenome-derived peptidase from a thermophilic environment. As PepBW1 is derived from an archaeal organism, it illustrates that screening in a heterologous host can be successful, even if the target gene originates from a different domain of life [56].

### 4.3. Ecology of Hot Springs

As most studies on ecology of hot springs are targeting the prokaryotic diversity, for example, via 16S rRNA gene or other marker gene analyses, little is known on the global relevance of these extremophilic communities. Burgess et al. (2011) studied two thermal pools in the Uzon Caldera by 16S rRNA gene analysis and related some community members to different archaeal and bacterial groups, which might play a role in cycling of C, N, and S [57]. 

The vital role of *Archaea* in N_2_ fixation and denitrification is well established [58]. The first step of nitrification, ammonium oxidation, was originally thought to be restricted to some *Proteobacteria* [59]. However, recent metagenomic studies provided evidence that *Archaea* are capable to oxidize ammonium to nitrate [58, 59]. Until recently, methanogenic *Euryarchaeota* were thought to be the only archaeal group of global relevance for element cycling. This presumption changed with the discovery of ammonia-oxidizing archaea [60], which are affiliated to the recently proposed phylum *Thaumarchaeota*. Members of this phylum contribute significantly to the global N cycle, as their high abundance and extremely low substrate threshold provides compelling evidence for a dominant role as ammonia oxidizers in open oceans [60]. In our study, we identified diverse thaumarchael groups in both investigated sediment samples. Thus, hot springs may also play a major role in the global N cycle.

## Figures and Tables

**Table 1:** Chemical analysis of the two investigated sediment samples (DM: dry matter, DIN: in accordance with the DIN (German Institute for Standardization) norm, VDLUFA: Association of German Agricultural Analytic and Research Institutes).

Element	Method	Mutnovsky (mg/kg DM)	Uzon Caldera (mg/kg DM)
Aluminum	DIN ISO 22036	22000	13000
Arsenic	DIN ISO 22036	21	590
Barium	DIN ISO 22036	20	120
Beryllium	DIN ISO 22036	<0.20	<0.20
Boron	DIN ISO 22036	<3.0	8.7
Cadmium	DIN ISO 22036	<0.10	<0.10
Calcium	DIN ISO 22036	23000	17000
Chromium	DIN ISO 22036	580	320
Cobalt	DIN ISO 22036	45	11
Copper	DIN ISO 22036	240	22
Iron	DIN ISO 22036	97000	27000
Lead	DIN ISO 22036	5.7	<2.0
Magnesium	DIN ISO 22036	2800	4200
Manganese	DIN ISO 22036	150	440
Nickel	DIN ISO 22036	270	150
Phosphorus	DIN ISO 22036	230	130
Potassium	DIN ISO 22036	120	530
Sodium	DIN ISO 22036	110	1200
Strontium	DIN ISO 22036	24	40
Titanium	DIN ISO 22036	1100	870
Vanadium	DIN ISO 22036	82	57
Zinc	DIN ISO 22036	73	42
pH (CaCl_2_)*	VDLUFA-Method A 5.1.1	6.4	6.8
TOC	DIN EN 13137	1.06	0.67

*pH values of the two sediments were measured in situ.

**Table 2:** Prokaryotic diversity and richness at 1, 3, and 20% genetic distance. Numbers of observed OTUs as well as Shannon and Chao1 indices were calculated with QIIME [16]. The maximal number of OTUs (*n*_max⁡_) was calculated using the Michaelis-Menten-fit diversity metrics implemented in the QIIME package. Coverage was determined by dividing the observed number of OTUs with *n*_max⁡_.

Sample	Observed OTUs			Max. OTUs (*n*_max⁡_)			Coverage (%)			Shannon index (*H*′)			Chao1		
	1%	3%	20%	1%	3%	20%	1%	3%	20%	1%	3%	20%	1%	3%	20%
	*Archaea*														

Mutnovsky	33	25	7	37.7	27.4	7.2	88	91.2	97.2	2.96	2.70	1.87	44.3	28.3	7
Uzon	13	11	5	14	11.6	5	93	94.8	100	1.83	1.67	0.86	13	11	5

	*Bacteria*														

Mutnovsky	17	12	10	18.2	12.3	10	93.4	97.6	100	2	1.81	1.65	32	15	13
Uzon	50	42	11	62.9	52.5	11.3	95.8	80.0	97.3	2.9	2.60	1.63	69.3	56.4	11.5

**Table 3:** Novel lipolytic and proteolytic enzymes and their closest relatives in the NCBI database.

Protein	Length (amino acids)	Closest similar protein, accession no. of similar protein	Corresponding organism	*E*-value	Amino acid homology to the closest similar protein (% identity)
PlpBW1	250	Patatin, YP_002729059	*Sulfurihydrogenibium azorense *Az-Fu1	9*e* − 111	1–250 (75%)
EstBW1	254	Alpha/beta hydrolase family protein, ZP_03857090	*Thermobaculum terrenum *ATCC BAA-798	4*e* − 61	2–251 (47%)
EstBW2	191	Hypothetical protein SULAZ_0137, YP_ 002728134	*Sulfurihydrogenibium azorense *Az-Fu1	2*e* − 91	1–188 (85%)
PepBW1	411	Subtilisin-like serine protease, YP_002428837.1	*Desulfurococcus kamchatkensis *	0	1–411 (98%)

**Table 4:** Relative activities of recombinant esterases in the presence of different chemical compounds. The effect of the additives was further tested for significance. Significant effects (*P* < 0.05) are written in bold type.

Detergent	Relative activity of PlpBW1 (%)	Relative activity of EstBW1 (%)	Relative activity of EstBW2 (%)
AgNO_3_	106.80 ± 16.06	**34.27 ± 1.66**	**163.17 ± 4.88**
CaCl_2_	**132.39 ± 9.63**	**85.35 ± 6.30**	97.10 ± 0.98
CoCl_2_	**131.17 ± 5.93**	104.94 ± 7.29	**64.00 ± 5.60**
CTAB	**274.39 ± 34.52**	**40.66 ± 6.67**	**8.11 ± 3.30**
CuCl_2_	125.48 ± 2.70	**81.03 ± 3.98**	94.20 ± 0.57
EDTA	119.45 ± 7.50	101.47 ± 10.75	96.03 ± 4.24
FeCl_3_	106.67 ± 8.94	**53.56 ± 12.56**	99.47 ± 5.01
KCl	124.50 ± 19.51	99.29 ± 12.11	96.40 ± 5.55
2-Mercaptoethanol	84.82 ± 7.31	**0.00 ± 0.00**	78.74 ± 19.95
MgCl_2_	**133.37 ± 7.25**	90.38 ± 13.06	101.54 ± 2.87
MnCl_2_	72.07 ± 2.31	82.14 ± 14.16	**48.90 ± 5.22**
NaCl	131.24 ± 15.17	98.59 ± 20.07	92.42 ± 3.60
NiSO_4_	121.86 ± 14.71	89.64 ± 4.60	**45.65 ± 8.03**
PMSF	77.49 ± 16.91	**0.08 ± 16.43**	**0 ± 4.16**
SDS	68.45 ± 17.38	**22.47 ± 9.64**	**39.49 ± 3.14**
Tween 80	**174.52 ± 2.29**	**60.70 ± 12.74**	**141.50 ± 4.94**
ZnCl_2_	**72.01 ± 1.77**	**59.26 ± 5.72**	**8.64 ± 1.71**

Abbreviations: CTAB: cetyltrimethylammonium bromide; EDTA: ethylenediaminetetraacetic acid; SDS: sodium dodecyl sulfate; PMSF: phenylmethylsulfonyl fluoride.
